# Variations in soil erodibility (K-factor) for the Chernozems depending on the method of texture determination

**DOI:** 10.1016/j.mex.2024.102876

**Published:** 2024-07-23

**Authors:** Mikhail Komissarov, Daria Fomicheva, Andrey Zhidkin, Anna Yudina

**Affiliations:** a V.V. Dokuchaev Soil Science Institute, Pyzhevskiy Pereulok 7, 119017 Moscow, Russia; b Ufa Institute of Biology UFRC RAS, Pr. Oktyabrya 69, 450054 Ufa, Russia

**Keywords:** Central Russian Upland, Erosion modeling, Revised universal soil loss equation, Soil texture, Laser diffraction, Peculiarities in calculation of RUSLE K-factor depending on method of Сhernozems texture determination

## Abstract

Soil erodibility (K-factor) is an important parameter in erosion modeling, is one of five factors of the Revised Universal Soil Loss Equation (RUSLE), and generally represents the soil's response to rainfall and run-off erosivity. The erodibility could be determined based on direct measurements of soil properties and mathematical calculations. In this study, the K-factor was calculated based on a formula from RUSLE, proposed by Renard et al. (1997). All input parameters: soil organic carbon (SOC), soil structure, and permeability classes were measured by one method, but particle size distribution – in two ways by sedimentation and laser diffraction methods to assess the impact the K-factor variability and the values of soil erosion rates. The 107 soil samples of Chernozems from Kursk Oblast (Russia) were studied. The texture for the most of samples was classified as silty loam in both analyses. However, the laser diffraction underestimates the clay content by an average of 13.2 % compared to the pipette method. The average K-factor estimated based on laser diffraction data was 0.050, and 0.034 t ha h ha^−1^ MJ^−1^ mm^−1^ – sedimentation method. Thus, depending on the method of soil texture analysis, the RUSLE calculated soil loss could underestimated/overstated by 32 % (or 4 t ha^-1^ yr^-1^ on average in the study site). Therefore, we propose a regression equation-based conversion method of laser diffraction data to sedimentation method data for Chernozems.

• The Laska-TM laser analyzer measured on ∼ 13 % less clay fraction (more on ∼ 8 % silt and ∼ 5 % fine sand) compared with sedimentation method data.
• For erosional researchers/modelers it is suggested to state the method of soil texture analysis (based on sedimentation law or laser diffraction) was used for RUSLE K-factor calculations.
• To convert K-factor values (for Chernozems) calculated and based on data of the sedimentation method to laser sedimentation – it suggested utilize the coefficient 1.47 (0.68 – vice versa).

## Graphical abstract

**Figure:**
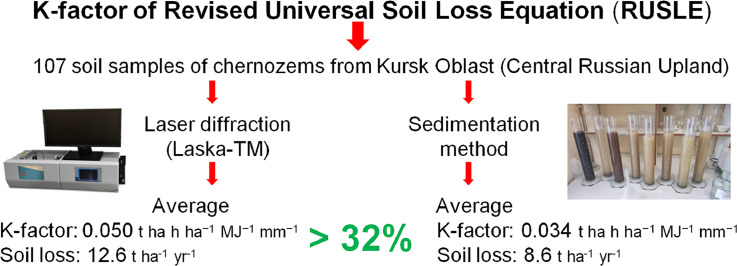

Specifications table

**Table:** 

Subject area:	Environmental Science
More specific subject area:	Soil erosion modeling
Name of your method:	Peculiarities in calculation of RUSLE K-factor depending on method of Сhernozems texture determination
Name and reference of original method:	Revised Universal Soil Loss Equation (RUSLE) K-factor K.G. Renard, G.R. Foster, G.A. Weesies, D.K. McCool, D.C. Yoder, Predicting soil erosion by water: A guide to conservation planning with the revised universal soil loss equation (RUSLE). USDA Agriculture Handbook No. 703, Washington, 1997.
Resource availability:	The data presented in this study are available upon request from the authors

## Background

*The following and existing approaches of the K-factor estimation currently are known:*

(i) The soil erodibility (K-factor) in field conditions is determined as the ratio of the average annual soil loss to the rainfall erosivity index (R-factor). Usually, it is provided on standardized runoff plots with the following parameters: 1) slope 9 %, length 22.1 m; 2) plot is fallow and tillage is up and down the slope and no conservation practices are applied (CP=1) [1].
(ii) In case of discrepancy runoff plot standard parameters (LC- and CP-factors differ from “standardized plot”), the K-factor could be determined for “any place” based on field observations and calculations using erosion models equations. For example, based on back calculation, the K-factor from the USLE [1] “in theory” is defined as:
  (1) K=A/R×L×S×C×P

where A is the measured mean annual soil loss in t ha^−1^ yr^−1^, R is the rainfall erosivity (MJ mm ha^−1^ h^−1^ yr^−1^), L is the slope length factor, S is the slope steepness factor, C is the crop cover factor, and P is the support practice factor.

(iii) To avoid the expensive and time-consuming measurements on runoff plots, many researchers have tried to “accelerate” the acquisition of soil erodibility data by rainfall modeling [[2], [3], [4]]. However, sometimes, it is problematic to estimate accurate values of the erodibility by rainfall simulations. Marques et al. [5] showed that a portable rainfall simulator applied to measure the K-factor tends to underestimate soil loss and sediment delivery predictions, and its performance is near satisfactory, being an alternative for data-scarce environments.
(iv) Another way to determine the K-factor is the use of nomograms. Wischmeier et al. [6] systematized a huge array of data from several thousand observations from runoff plots and developed a soil erodibility’ nomogram. However, using the nomogram in some regions is not recommended because it usually overestimates K-factor values [7,8]. Wischmeier's nomogram relationship was derived from 55 surface soils in the Midwestern USA, which were mostly (81 %) medium-textured [9]. When the nomogram relationship is applied to soils with characteristics similar to those in that part of the USA, a close correlation has been found between predicted and measured values. However, difficulties arise and poorer predictions are obtained when it is necessary to extrapolate the nomogram values [10].
(v) Recently, the K-factor is more often determined based on formulas for calculating this indicator based on soil properties. For example, soil erodibility could be estimated based on data on soil organic carbon (SOC) content, particle size fractions, type of clay [11], aggregate stability [12,13], soil cohesion [14], saturated conductivity [15], the number of drop impact [16], the mean weight diameter of soil aggregates [17], and penetration resistance [18]. A significant number of developed equations for K-factor calculation based on soil properties are given in the review [19].

Among the mathematical equations, the most common and widely used is the equation of Renard et al. [9] (formula 2), since it is adapted for most soil types. However, the resulting K-factor value may vary depending on the method of measuring some input parameters (particle size distribution, SOC, etc.). For soil texture determination the methods based on sedimentation law and laser diffraction are used. However, there is a significant difference between the results of these groups of methods – the content of fine particles obtained by laser diffraction is always lower than by methods based on the sedimentation law [20]. Therefore, in this study we analyzed how the method of texture measuring impact on the K-factor value and final erosion rates by applying of RUSLE model for Сhernozems of the Central Russian Upland.

## Method details

### Description of study site, field and laboratory works

The study site is located in the south-western part of the Central Russian Upland (Kursk Oblast, Russia). The area is characterized by undulating topography with the domination of gentle (2° to 5°) convex slopes of moderate length (200–500 m). The climate of the Kursk Oblast is temperate continental, with moderately cold winters and warm summers (*Dfb* according to the Köppen-Geiger classification [21]). The average annual air temperature in January is –8.6 °C, in July – 19.3 °C. The average annual precipitation is 550–600 mm, and about 70 % of precipitation falls from April to October. Stable snow cover forms in the 1st half of December and disappears in the 1st decade of April; its thickness is 20–30 cm. The growing season varies from 180 days in the north to 195 days in the south. Such conditions contributed to the formation of the most fertile soils in the world – Chernozems.

**Fig. 1:**
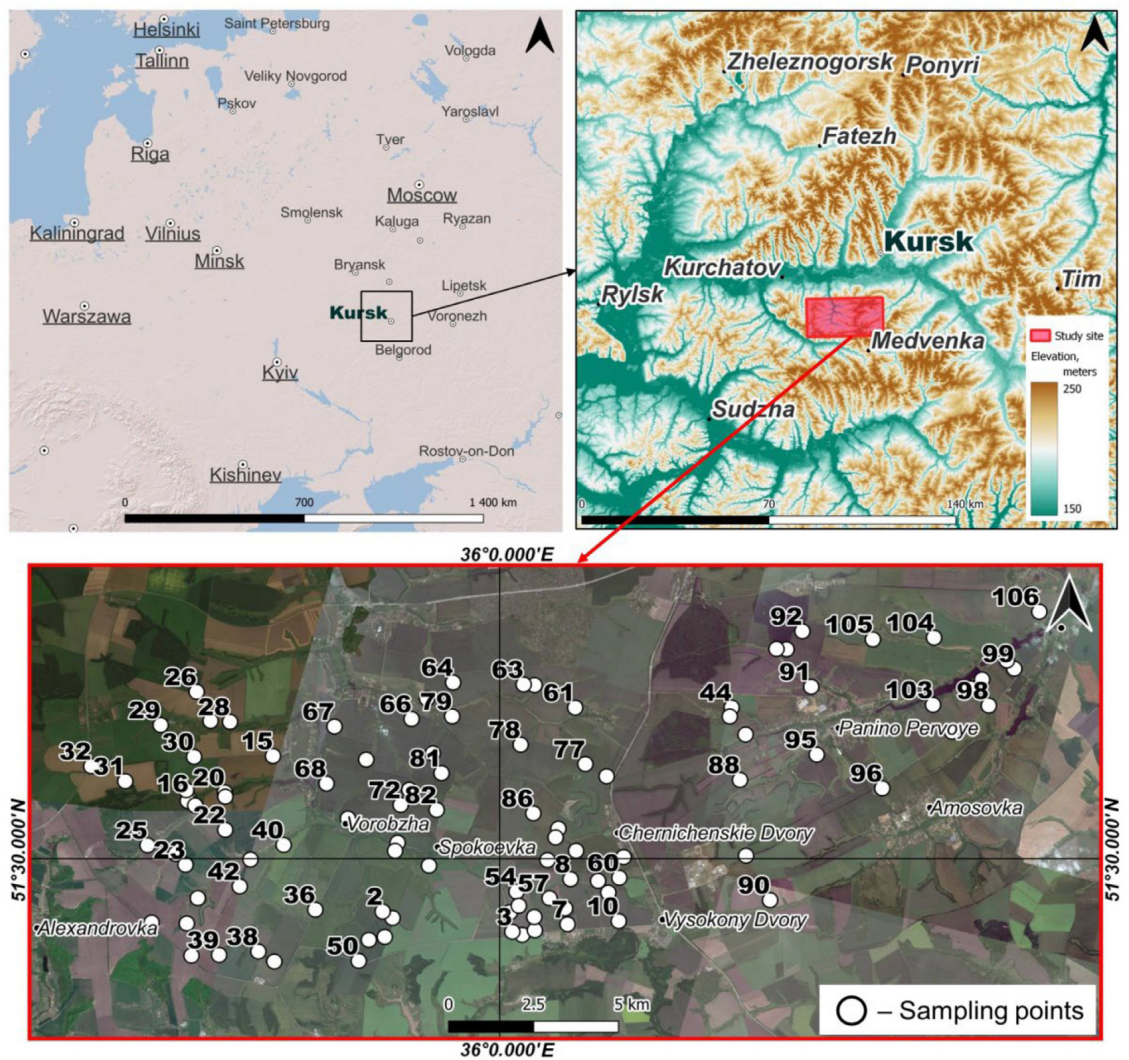
Location of sampling points.

107 soil samples were randomly collected from a topsoil (0–25 cm) of croplands on an area of about 10,000 ha (Fig. 1). For soil sampling a hand Edelman drill (Royal Eijkelkamp, Netherlands) with diameter equal to 5 cm was used. Sampling was carried out in a mixed sample of three repetitions. The location of the studied points was recorded using a GPS navigator. Additionally, at each studied point, the drilling (as alternative way of soil profiles excavating) was provided by a sampler to the illuvial horizon (B) on average to a depth of 1 m. This was done for the soil diagnostics (subtype) determination.

In the laboratory, the soil samples were dried in an oven (at 90 °C) to constant weight and prepared (crushed and sieved) for ensuing analyses. The SOC content in samples was determined by the Tyurin method [22] using spectrophotometer Specord M40 (VEB Carl Zeiss, Germany).

Analysis of the soil texture by laser diffraction method was performed on a laser particle size analyzer Laska-TD (Biomedical Systems, Russia). The analyzer measures the particles from 1 to 1,000 µm by one scan laser (670 nm). Pre-measurement preparation of one sample included: i) a 5-minute treatment of the undisturbed soil with distilled water in an ultrasonic bath Vilitek VBS-1H (Vilitek, Russia; at frequency of 40 kHz), ii) separation of soil particles >0.1 mm by wet sieving, then iii) a 3-fold analysis for a suspensions (separated soil fraction <0.1 mm) in concentrations of 80, 50 and 30 % were provided. The Atterberg soil particle size classification [23] was used: clay (<0.002 mm), silt (0.002–0.05 mm), and very fine sand (0.05–0.1 mm). It is worth noting that conversion of the granulometric composition results into the unified international Atterberg classification is necessary for subsequent calculations of soil erodibility (K-factor) (see formula (2)).

Analysis of the soil texture by sedimentation (pipette) method was carried out according to GOST 12536 [24]; the procedure's details also are explained in Vadunina and Korchagina [25]. According to this methodology, the measurement of the fractions is performed according to the Kachinsky soil particle size classification [26]: clay (<0.001 mm), very fine silt (0.005–0.001 mm), medium silt (0.01–0.005), very fine sand (0.05–0.01 mm), fine sand (0.25–0.05 mm), and coarse sand (1.0–0.25 mm). To convert from the Kachinsky classification to the international (Atterberg) one, we used the method proposed by Shein [27]. The method is based on the approximation that the cumulative distribution curve of soil particles in the range of 0.001–0.005 mm is close to a linear function. Fraction values <0.002 mm and 0.002–0.05 mm were recalculated proportionally. Fraction values <0.1 mm were used in the calculations and their total distribution was taken as 100 %.

When the K-factor in each study point was calculated based on RUSLE Eq. (2) proposed by Renard et al. [8].

(2) $$K=[\left(2.1\;\times 10^{-4}M^{1.14}\left(12-OM\right)+3.25\left(s-2\right)+25\left(p-3\right)/100\right]\;\times \;0.1317$$

where M – the textural factor with *M* = (m_silt_ + m_vfs_) × (100 − m_c_); m_c_ [%] – clay fraction content (<0.002 mm); m_silt_ [%] – silt fraction content (0.002–0.05 mm); m_vfs_ [%] – very fine sand fraction content (0.05–0.1 mm); OM [%] – organic matter content; s – the soil structure class (*s* = 1: very fine granular, *s* = 2: fine granular, *s* = 3, medium or coarse granular, *s* = 4: blocky, platy or massive); p – the permeability class (*p* = 1: very rapid, …, *p* = 6: very slow).

To determine the influence of K-factor method estimation on the values of the mean annual soil erosion rates we applied the RUSLE model. We used the same input parameters, except for erodibility. The R-factor was taken equal to 320 MJ mm ha^−1^ h^−1^ yr^−1^ according to [28]. The C-factor was assumed to be 0.40 according to data on the portion of crops in the crop rotation and the agroerosion index of crops in this zone according to [29]. The LS factor was automatically calculated in the RUSLE program based on the digital terrain model and equations [30]. The digital terrain model was obtained based on the open access DEM, STRM data with the cell size 30 × 30 m. The soil structure and permeability classes were taken based on the soil texture class [31], 2 and 3, respectively. The erosion modeling input parameters were verified on a small catchment within the study site [32]. The statistical analyses were provided in the Statistica 12.0 (TIBCO Software Inc., USA).

## Results and discussion

**Fig. 2:**
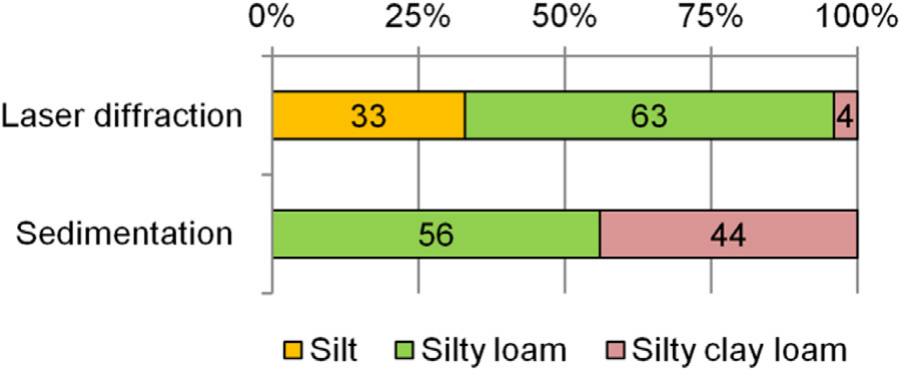
Distribution of studied soil samples by texture class depending on the method used.

According to the field survey, all sampling point belongs to the Chernozems (and presented by Haplic and Luvic Chernozems according to the WRB classification [33]). A morphological description of representative soil cross-section is following: A (0–50 cm) – dark gray, dry, fine-grained, loam (texture was determined by feel), slightly compacted, plant roots, the transition is smooth in color; AB (50–70 cm) – dark gray, moist, granular-lumpy, silt loam, moderately compacted, rarely plant roots, the transition is smooth in color; B (70–… cm) – dark gray, wet, medium-prismatic (in some places – structureless), clay loam, compacted, rarely small plant roots, carbonate inclusions, effervescence with 10 % HCl. The level of effervescence with 10 % HCl depended of Chernozem type: in Haplic was 30–115 cm, and in Luvic – 55–150 cm. The results of the laser analyzer showed, that according to the international classification of soil texture [34], the soils are mainly classified as silty loam (63 %); silt (33 %), and silty clay loam (4 %) also found (Fig. 2). Using the sedimentation method, soil samples have a silty loam (56 %) and a silty clay loam texture (44 %).

**Fig. 3:**
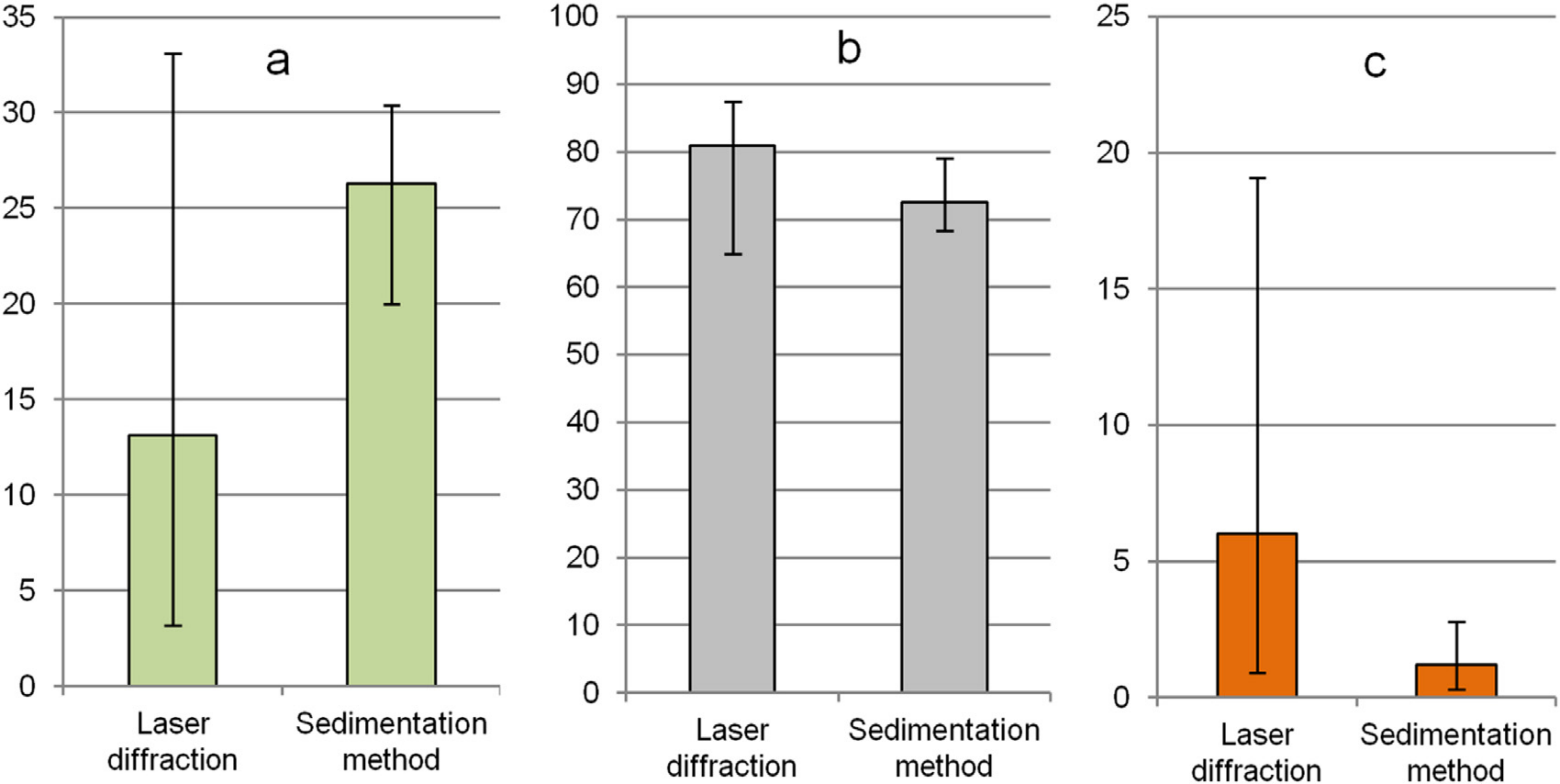
Contents (%) of clay (<0.002 mm) (a), silt (0.002–0.05 mm) (b) and very fine sand (0.05–0.1 mm) (c) fractions in studied Chernozems determined by different soil texture analysis methods.

**Fig. 4:**
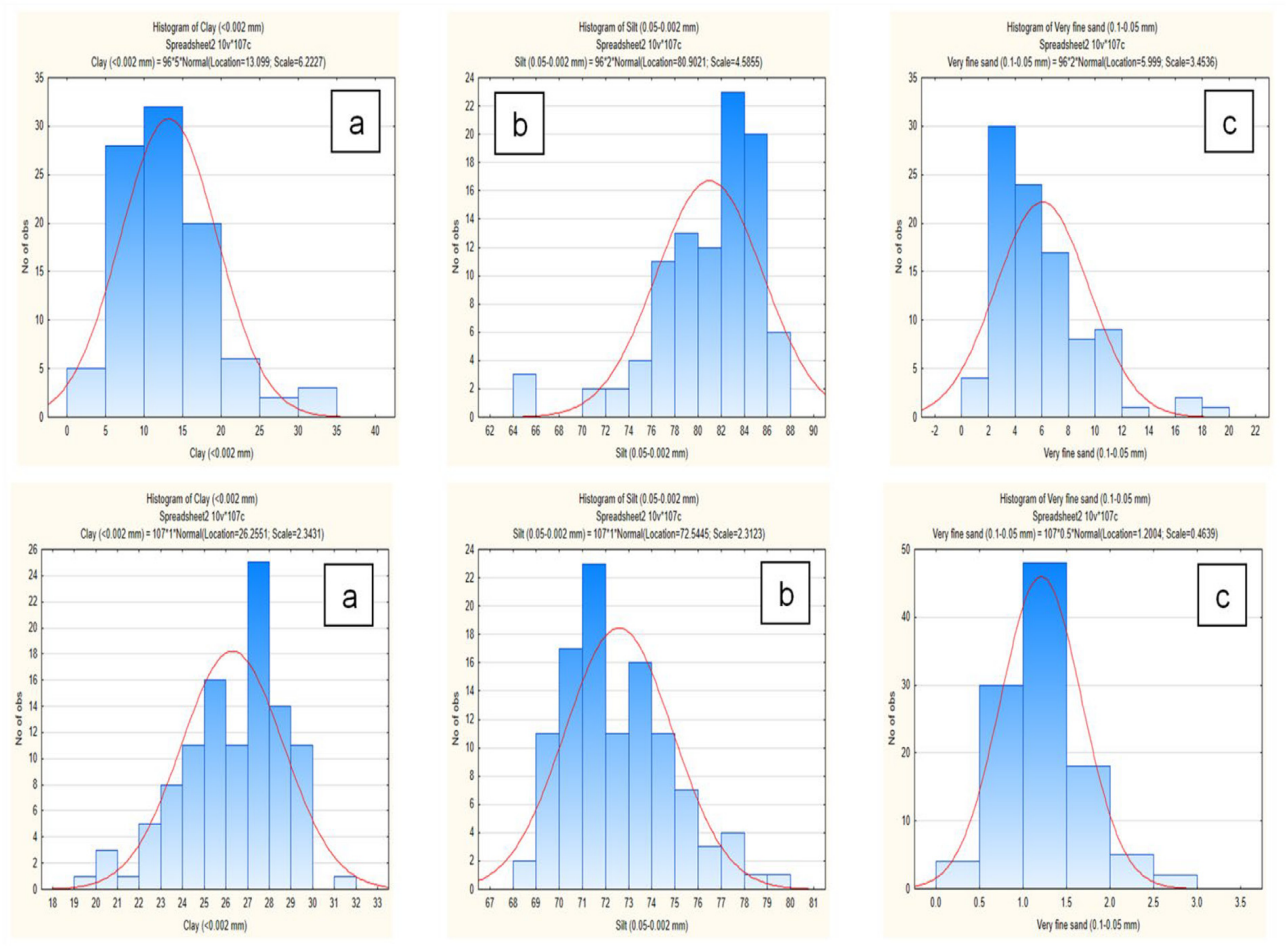
Distribution of samples (numbers, Y-axis) and particle size fractions (%, X-axis) – clay (<0.002 mm) (a), silt (0.002–0.05 mm) (b) and very fine sand (0.05–0.1 mm) (c). The top row is the laser diffraction, the bottom row is sedimentation method.

In the category of fine particles, samples with a silt content in the range from 82 to 86 % predominate. When using the sedimentation method, the dominant samples with a silt content shift to the boundaries of 70–72 %. In laser analysis, 1/3 of the samples are characterized by the sand content (very fine sand) of 2–4 %; under sedimentation, for almost half of the samples, the sand content is of 1.0–1.5 %. The average clay content (<0.002 mm) determined using a laser analyzer is 13.2 % lower than using the sedimentation method (Fig. 3a); silt (0.002–0.05 mm) is higher by 8.4 % (Fig. 3b), very fine sand (0.05–0.1 mm) – higher by 4.8 % (Fig. 3c). The largest proportion of samples (about 1/3), when using the laser diffraction method, have a clay content of 10–15 %, while when using sedimentation, about quarter of the samples have a clay content of 27–28 % (Fig. 4a).

Numerous works [27,35,36] show that sedimentation method give higher values of fine fractions (clay and silt) due to the uneven distribution of solid phase density in different granulometric fractions. On the one hand, the laser diffraction method measures particle sizes “physically” due to refractions of the laser beam; while in the sedimentation method, the laws of sedimentation, in particular the Stokes law, are used in the calculations of fine fractions, and sieve measurements for large fractions. Both methods have both advantages and disadvantages: laser diffraction is fast, but can produce minor errors; sedimentation is labor intensive. Both methods characterize general trends in the distribution of soil particles.

**Fig. 5:**
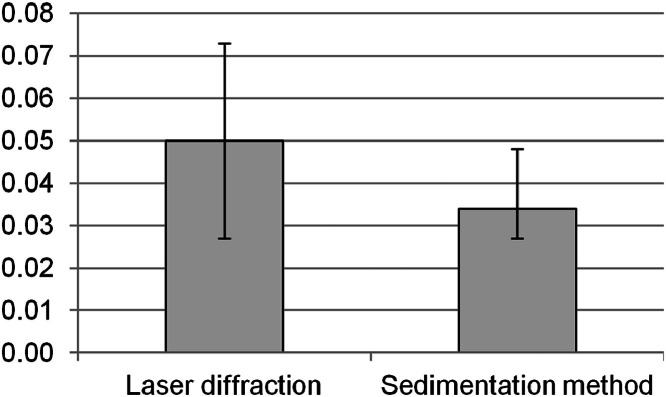
K-factor values (t ha h ha^−1^ MJ^−1^ mm^−1^) determined by different methods.

About ¼ of the samples measured by laser diffraction method have a K-factor value of 0.050–0.055, and about half in the range of 0.040–0.055 t ha h ha^−1^ MJ^−1^ mm^−1^ (Fig. 5). The average K-factor value based on particle size distribution data obtained using the laser diffraction was 32 % higher (*p* < 0.001) than using the sedimentation method.

**Fig. 6:**
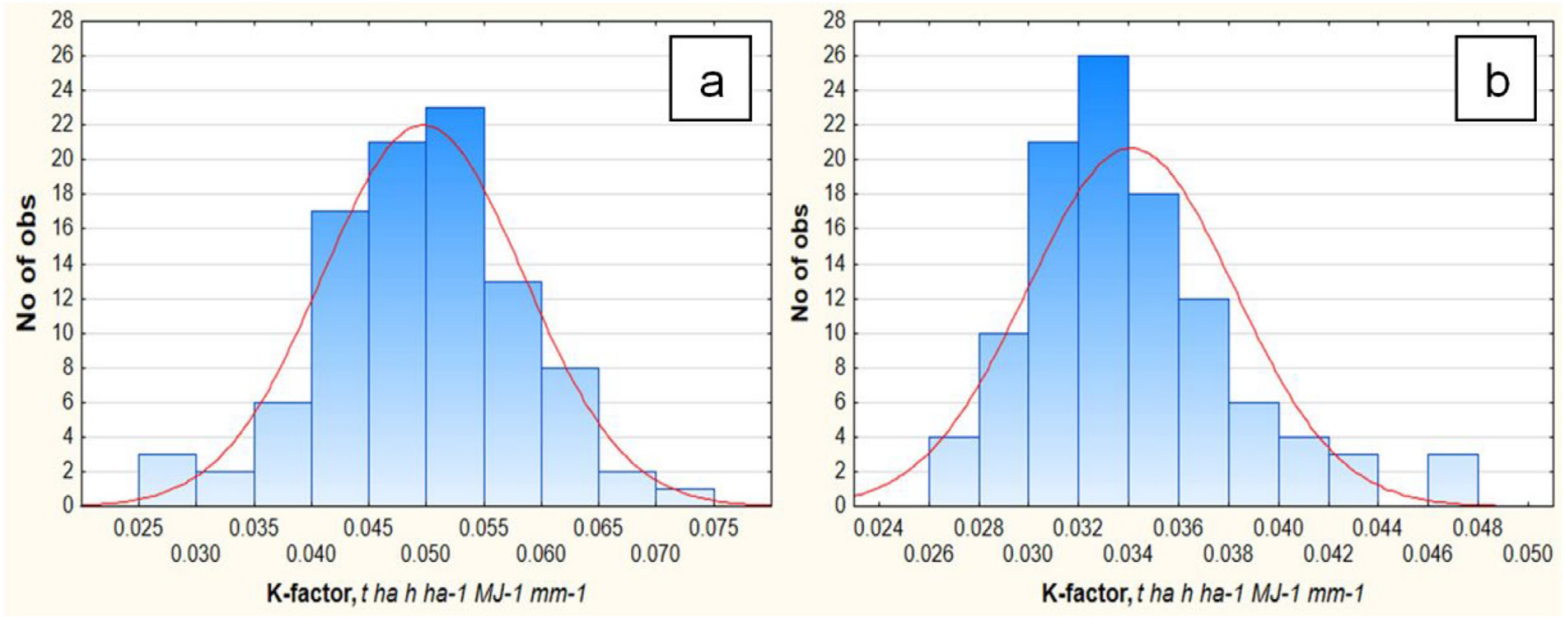
Distribution of K-factor values (X-axis)/samples (numbers, Y-axis) depending on the method of soil texture analysis: a – laser diffraction, b – sedimentation.

**Fig. 7:**
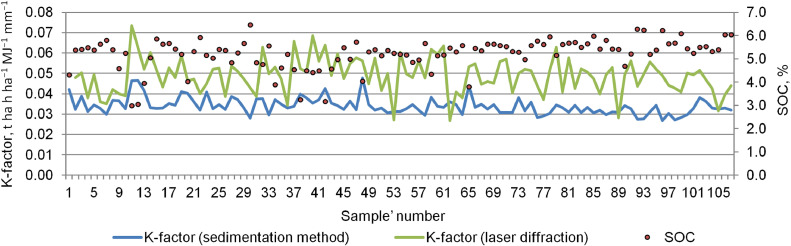
Relationship between K-factor and soil organic carbon (SOC) using different methods for determining the granulometric composition.

With the sedimentation method, half of the samples are distributed in the range 0.030–0.036 t ha h ha^−1^ MJ^−1^ mm^−1^. No definite relationship was identified in the uneven distribution of K-factor values obtained by different methods (Fig. 6). With a decrease in the SOC content, the differences in K-factor values become insignificant (*p* < 0.001) (Fig. 7). However, Fomicheva et al. [37] studying Chernozems of Oryol Oblast (borders with Kursk Oblast in the south) found that with an increase in the degree of soil erosional degradation and a decrease in the SOC content, the mean erodibility increases up to 0.057 t ha h ha^−1^ MJ^−1^ mm^−1^ (in strongly eroded Chernozems). The mean values of the K-factor of Chernozems calculated based on sedimentation method vary from 0.034 to 0.042 t ha h ha^−1^ MJ^−1^ mm^−1^, that is similar to our results obtained using the same method. Thus, erodibility of Chernozems is characterized by a close range of values regardless of their location. It is interesting to note, that the values of erodibility within Central Russian upland according to Gupta et al. [38] is lower in comparing with our results, and vary between 0.025 to 0.04 t ha h ha^−1^ MJ^−1^ mm^−1^.

There are few studies evaluating the effect of different measurement methods on the K-factor value. For example, Centeri et al. [39] studying Hungarian soils conclude, that the method of particle size measurement does affect on soil erodibility factors and thus, also on the amount of the calculated soil losses, even though there were no analyses of significance on the soil erodibility and soil loss calculations. The differences between the amounts of soil loss calculated with the measured particle size classes resulted in a very small (0.4 %) difference between the smallest and the greatest amount of soil loss. The highest difference of the measured values was 6.1 %, which can also be regarded as fairly low. However, if taking into account the soil loss and not the percentage – the amount of soil loss with the given parameterization is quite great, exceeding 70 t ^−1^ ha^-1^ y^-1^. In our research, in the case of using the K-factor obtained by laser diffraction method and applying RUSLE model the average soil loss for the studied area amounted 12.6 t ha^-1^ yr^-1^, while with the sedimentation method, it was lower by 32 % (8.6 t ha^-1^ yr^-1^).

## Method validation

In present study, we suggest to researchers indicate in scientific works (articles, report, etc.) which method was used for RUSLE K-factor calculations, because related with this variations in soil erodibility have an great impact on final soil loss estimations under modeling. Undoubtedly and ideally is provide a validation on standardized runoff plots, and then summarize which method of soil texture determination is more correct for K-factor calculations; however such field works required a long-term observations and labor intensive. Another way is compare and validates result of erosion modeling (with K-factor obtained by different methods) with field erosion data. However, even just field methods (soil-profile truncation, radiocesium technique, etc.) provide errors of 5–40 % in soil erosion rates estimation [40]. Thus, for validation and selection of the “best” RUSLE K-factor estimation method based on soil texture, we will search the data from standardized runoff plots for Chernozems in the Central Russian Upland or will provide such measurements.

## Limitations

Possible limitations are mainly related to the methods for determining input parameters of RUSLE K-factor formula (SOC, soil structure and permeability classes) and equipment. For example, for the SOC content determination we used Tyurin approach [22], since other methods, e.g. loss on ignition (LOI) could provide other results, and therefore impact on estimated final K-factor values. Other limitation related to location of Chernozems, for example, we cannot guarantee that our suggestions will be correct on Chernozems with other formation conditions (over-moistened, with very low or very high SOC content, in regions and/or countries different in climate from the Central Russian Upland). During K-factor calculations we used a soil structure code and soil permeability code based on soil texture class [31]; the convertation proposed by Bagarello et al. [41] is not studied in this research, thus we cannot state how it influence on K-factor calculations. In this study we tested Laska-TM laser analyzer, probably other devices with different technical characteristics may show different results. For example, diffractometer Laser Particle Sizer Analysette 22 (Fritsch GmbH, Germany) contains a helium-neon laser below 5 mW and a wavelength of 655 nm and measuring range 0.1–670 µm. The Mastersizer 3000 (Malvern Instruments, UK) measure particle size distributions from 10 nm up to 3.5 mm; 40x wavelength (25 µm when a He-Ne laser is used). The number of scanning lasers propably also could have effect on measurement results. Also, it should be noted to limitation related to using of Wischmeier's nomogram, which work properly if silt and very fine sand content not exceeds 70 %. In present study, some of samples have a higher content, however in earlier study on Chernozems in the Central Russian Upland [39] we provided the verification and validation of RUSLE erosion modeling (even with high silt and very fine sand content during K-factor calculations) and results were similar/close with other methods of soil loss estimation.

## Conclusion

With laser diffraction, compared to the sedimentation/pipette method, the particle size distribution has ∼ 13 % less clay fraction, more ∼ 8 % of silt, and ∼ 5 % of fine sand. The soil texture with both methods is predominantly characterized as silty loam. The K-factor values calculated on the basis of granulometric composition using a sedimentation method on average was 0.034 t ha h ha^−1^ MJ^−1^ mm^−1^ (ranged from 0.029 to 0.049) and it on 32 % lower compared with the laser analyzer data. Consequently, the average soil loss calculated by applying RUSLE also varied depending on the method of texture determination: it was 12.6 t ha^-1^ yr^-1^ under laser diffraction and 8.6 t ha^-1^ yr^-1^ – sedimentation method. The erodibility of studied chernozems is close to the values that were previously established for the Kursk and Oryol Oblasts, thus it could be assumed that K-factors of Chernozems are the same regardless of their location. When calculating the RUSLE K-factor, it is necessary to indicate which method was used to analyze the soil texture.

## Ethics statements

No ethic statements to declare.

## CRediT authorship contribution statement

**Mikhail Komissarov:** Writing – original draft, Writing – review & editing, Investigation, Data curation, Software. **Daria Fomicheva:** Software, Data curation, Investigation, Visualization, Validation. **Andrey Zhidkin:** Supervision, Conceptualization, Methodology, Investigation, Writing – review & editing. **Anna Yudina:** Writing – review & editing.

## Declaration of competing interest

The authors declare that they have no known competing financial interests or personal relationships that could have appeared to influence the work reported in this paper.

## Data Availability

Data will be made available on request.
